# Hereditary hemochromatosis and JAK2‐positive polycythemia vera

**DOI:** 10.1002/ccr3.4907

**Published:** 2021-10-04

**Authors:** Ahmed Radwan, Ibraheem Othman

**Affiliations:** ^1^ College of Medicine University of Saskatchewan Saskatoon SK Canada; ^2^ Allan Blair Cancer Centre College of Medicine University of Saskatchewan Regina SK Canada

## Abstract

A 59‐year‐old man was diagnosed with JAK2‐positive polycythemia vera. Subsequently, further laboratory testing revealed elevated ferritin and iron saturation. Genetic testing for HFE gene mutation screen revealed that the patient was positive for heterozygous C282Y mutation. The patient was ultimately diagnosed with both polycythemia vera and hereditary hemochromatosis.

## Graphical abstract

This case report highlights the crucial role of maintaining clinical suspicion for hereditary hemochromatosis in patients with polycythemia vera and vice versa.

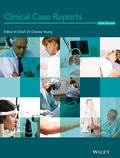

## 1 BACKGROUND

Hereditary hemochromatosis is an autosomal recessive disease characterized by increased intestinal absorption of iron, leading to excessive iron deposition in various organs and tissues. It is caused by a mutation in the HFE gene. The two most common HFE gene mutations are C282Y and H63D. 80%‐90% of patients are homozygous for C282Y mutation, with heterozygosity for C282Y and H63D being the second most common.^1^

Polycythemia vera is a myeloproliferative disorder characterized by clonal proliferation of the myeloid cell line. JAK2 mutation is proposed to be the cause in 95% of patients.^2^ Polycythemia vera increases the risk of thrombotic events with estimates that 34%‐39% of patients have major thrombotic events at the time of diagnosis. Thrombosis can sometimes involve the portal, hepatic (Budd‐Chiari Syndrome), splenic, and mesenteric veins.^3^

## 2 CASE REPORT

This is a case report of a 59‐year‐old man who in 2016 presented with joint pains affecting the ribs, lower back, hips, and knees. He denied fever, night sweats, loss of weight, or loss of appetite. Review of systems was unremarkable. Physical examination was normal. He did not have any skin discoloration or signs of plethoric facies. Complete blood count showed a hemoglobin of 18.2 g/dL, hematocrit of 54.2%, red blood cell count of 6.13 × 10^12^/L, mean corpuscular volume of 88.5 fl, and platelet count of 456 × 10^9^/L. Erythropoietin was 3.2 IU/L which is within the normal range, and peripheral blood smear showed normal morphology. JAK2 mutation was sent, and results came back positive. The patient was then diagnosed with JAK2‐positive polycythemia vera according to the World Health Organization (WHO) Diagnostic Criteria for Polycythemia Vera.^4^ He was then started on phlebotomy and aspirin therapy. However, the patient was also found to have a ferritin of 603 μg/L, iron of 48.5 μmol/L, and iron saturation 83%. Subsequently, genetic testing was sent for HFE gene mutation, and the patient was found to be positive for heterozygous C282Y mutation. The patient was also found to have a strong family history for hereditary hemochromatosis in one brother and two sisters.

Ultimately, he was diagnosed with both polycythemia vera and hereditary hemochromatosis. He was then recommended to continue aspirin and phlebotomy to maintain a hematocrit value below 45%. Cytoreductive therapy with hydroxyurea was not required because the patient had low risk for thrombotic events because of his relatively young age and absence of prior history of thrombotic events.

## 3 DISCUSSION

Initially, it was thought that there is no association between polycythemia vera and hereditary hemochromatosis. In 2002, a study analyzed C282Y and H63D mutations in 232 patients with different hematological disorders and found no significant associations, particularly with polycythemia vera.^5^ In 2004, another study analyzed 52 patients with polycythemia vera for hereditary hemochromatosis and found no association between both conditions.^6^ However, in 2016, a case report was published detailing an association found between hereditary hemochromatosis and polycythemia vera in a 75‐year‐old woman. The patient had a past history of hereditary hemochromatosis and was receiving phlebotomies for 15 years. Upon presentation to cancer clinic, she was found to have elevated hemoglobin, low erythropoietin and ultimately, tested positive for JAK2 mutation.

To our knowledge, this is the second reported association between polycythemia vera and hereditary hemochromatosis after the one reported in 2016.^7^ While hereditary hemochromatosis and polycythemia vera seem to be unrelated, this case provides the basis that such conditions can co‐exist, which may lead to drastic complications in care management. As a result, we recommend that patients with polycythemia vera be screened for hereditary hemochromatosis and vice versa. This case report highlights the crucial role of maintaining clinical suspicion for hereditary hemochromatosis in patients with polycythemia vera.

## CONFLICTS OF INTEREST

The authors declare that they have no conflicts of interest to disclose.

## AUTHOR CONTRIBUTIONS

Ibraheem Othman and Ahmed Radwan involved in manuscript design. Ahmed Radwan involved in data analysis. Ahmed Radwan involved in manuscript written, with revision from Ibraheem Othman. Ibraheem Othman involved in final approval.

## ETHICS APPROVAL

Exempted.

## CONSENT

Published with written consent of the patient.

## Data Availability

The datasets generated during the current study are not publicly available for confidentiality reasons but are available from the corresponding author on reasonable request.
